# Hemodynamic and autonomic response to acute hemorrhage in streptozotocin-induced diabetic rats

**DOI:** 10.1186/1475-2840-9-78

**Published:** 2010-11-25

**Authors:** Aiji Boku, Mitsutaka Sugimura, Yoshinari Morimoto, Hiroshi Hanamoto, Hitoshi Niwa

**Affiliations:** 1 Department of Dental Anesthesiology Osaka University Graduate School of Dentistry, Suita, Japan

## Abstract

**Background:**

The various autonomic control systems lead to characteristic changes in heart rate (HR) and blood pressure (BP) during acute hemorrhage. However, cardiovascular autonomic neuropathy due to diabetes mellitus may interfere with the normal compensation for hemorrhage.

**Materials and methods:**

A controlled graded bleeding (6 - 36% loss of estimated total blood volume: ETBV) was performed in streptozotocin-induced diabetic rats (STZ rats) under a conscious state. Hemodynamic and autonomic responses to acute hemorrhage were examined using analysis of BP-HR variability. The effects of dextran treatment after hemorrhage were also examined.

**Results:**

A significant reduction in mean arterial pressure began at 12% ETBV loss in STZ rats and 18% in the control rats, respectively. When blood loss reached 18% of TEBV, the decrease in HR was prominent in STD rats due to the activation of a parasympathetic drive, as indicated by the increase in high frequency (HF; 0.75~3.0 Hz) power in HR variability, while in the control rats this response was not observed. The administration of dextran prevented the activation of the parasympathetic drive in STZ rats during hemorrhaging. In the control rats, the dextran treatment sustained the initial increase in HR with reduced HF power in HR variability.

**Conclusion:**

STZ rats showed different hemodynamic and autonomic responses to acute hemorrhage from the control rats. STZ rats were prone to develop bradycardiac hypotension characterized by marked parasympathetic activation during hemorrhaging. This finding suggests enhancement of the Bezold-Jarisch reflex in STZ rats. Dextran treatment to maintain a normovolemic hemorrhage state inhibits this reflex.

## Introduction

Cardiovascular autonomic neuropathy (CAN) is a major complication of diabetes mellitus and the most clinically important form of diabetic neuropathy. CAN is associated with abnormalities in heart rate control and vascular dynamics. Clinical manifestations of CAN include orthostatic hypotension, resting tachycardia, exercise intolerance, intraoperative cardiovascular liability, and silent myocardial infarction [1]. Furthermore, it is known that CAN induces lethal cardiac arrhythmia from prolongation of the QT interval and can be a cause of sudden death.

Patients with CAN require close perioperative anesthesia supervision. Compared with non-diabetic patients, diabetic patients undergoing general anesthesia may experience a greater degree of reduction in heart rate (HR) and blood pressure (BP) during induction of anesthesia [2]. It has been demonstrated that intraoperative vasopressor support was needed more often in diabetic patients with CAN than in those without [3,4].

Acute bleeding is a major factor affecting perioperative circulatory dynamics. The classical description of the response to acute hemorrhage involves a sustained increase in sympathetic activity to maintain arterial BP. However, recent studies of the effects of acute hemorrhage have clearly demonstrated a biphasic hemodynamic response [5,6]. During mild hemorrhage, BP is well maintained by an increase in vascular resistance and HR. A baroreceptor-mediated sympatho-excitatory response is evident in this initial phase. When the central blood volume is critically reduced by more than 30% of ETBV, a second phase develops suddenly. This phase is characterized by withdrawal of sympathetic drive and activation of parasympathetic drive. A severe fall in BP associated with bradycardia develops. Blood volume and cardiac filling become critically reduced, which activates the sympatho-inhibitory cardiodepressor reflex (Bezold-Jarisch (B-J) reflex) [7-9]. This reflex probably originates in the heart and is triggered by activation of myocardial mechanoreceptors, which are stimulated by vigorous contractions around a nearly empty left ventricular cavity. As a result, vagal afferents inhibit central cardiovascular centers, overrule their baroreflex-mediated activation, and deactivate the sympathetic nervous system. The B-J reflex is thought to be a protective response, defending the heart against ischemia during severe hypovolemia by reducing its workload through induction of bradycardia, negative inotoropy, and afterload reduction.

In diabetic patients with CAN, this compensation mechanism may not work adequately because some evidence suggests that both the sympathetic and parasympathetic nervous system are impaired in patients with insulin-dependent diabetes mellitus [1,10,11]. However, little is known about autonomic and hemodynamic responses to acute hemorrhage in diabetic patients with CAN.

In the present study, we explored hemodynamic and autonomic responses to acute hemorrhage in streptozotocin-induced diabetic rats (STZ rats). The present study was designed to minimize handling-induced stress and well-known cardiovascular and autonomic effects elicited by commonly used anesthetic agents.

Cardiovascular autonomic function was assessed by using spectral analysis of BP and HR variability. Using this method, we could evaluate autonomic function with minimum intervention throughout the study. Data analysis was performed with wavelet transformation because this method has a high time-resolving power. We evaluated efferent sympathetic activity using the low frequency (LF) component of systolic blood pressure (SBP) variability [12]. The LF component of SBP (SBP-LF) increases in conditions associated with various sympathetic activations [13-15]. Furthermore, SBP-LF is positively correlated with muscular sympathetic nerve activity, providing more direct support for the concept of using changes in the SBP-LF as a marker of changes in sympathetic efferent activity in the peripheral vasculature [16]. Therefore, we believe that analyzing SBP-LF can be used to assess sympathetic activity during acute hemorrhage. Power-spectral analysis of HR variability is routinely used as a noninvasive means of quantifying cardiac autonomic input. The high frequency (HF) peak represents respiratory sinus arrhythmia and is a reliable indicator of parasympathetic efferent activity. The ratio of LF to HF in HR variability was also calculated as an index of the autonomic nervous system balance [17]. Although the plasma catecholamine concentration is a useful indice of sympathetic activity, it is not so sensitive to changes in sympathetic nervous activity, so spectral analysis of BP and HR variability were used in the present study [18,19].

Here, we aimed to investigate the effects of diabetic CAN on response to acute hemorrhage. To this end, we studied hemodynamic and autonomic responses to acute hemorrhage in STZ rats. Using conscious unrestrained rats and spectral analysis of BP and HR variability enabled us to the isolate influence of acute hemorrhage.

## Materials and methods

The study protocol for animal experiments was approved by the Institute of Experimental Animal Sciences, Osaka University Graduate School of Dentistry, and carried out in accordance with the National Institutes of Health Guide for the Care and Use of Laboratory Animals (NIH Publications No. 80-23), revised in 1996. All efforts were made to minimize the number of animals used and their suffering.

### Animal preparation

Experiments were performed on male Wistar rats weighing between 300 and 350 g housed in individual cages with free access to water and standard rat food. After an overnight fast (about 12 hours), diabetes was induced by a single intraperitoneal injection of streptozotocin (STZ; 65 mg/kg). Five days after STZ administration, blood was obtained from a tail vein to determine blood glucose and ketone levels using a MediSence Precision Xtra™(Abbott Japan CO., LTD). Only those rats with plasma blood glucose concentrations of more than 300 mg/dl and a ketone body level of more than 0.5 mmol/l were considered diabetic and included in our study.

Eleven days after STZ administration, the diabetic rats were surgically prepared with catheters under anesthesia with intraperitoneal pentobarbital. A polyethylene catheter was inserted into a femoral artery for arterial pressure measurement and blood withdrawal. Another catheter was inserted into the left jugular vein for drug administration. These catheters were tunneled subcutaneously to exit from the neck. To prevent catheter obstruction, heparin-containing saline was injected every 12 hours until the experiment day. Rats were allowed 3 days to recover from surgery. As a control group, rats without STZ administration received the same operation (control rats).

### Analysis of autonomic nervous system activity

The arterial catheter was connected to a pressure transducer and a blood pressure waveform was amplified. The signal passed through an A/D converter (KPCMCIA-16AI-C, KEITHLEY) at a sampling frequency of 1,000Hz was stored continuously on a personal computer for later analysis. The mean arterial pressure (MAP) and SBP were recorded from artifact-free digitized signals using commercially available software for rats (Fluclet™, Dainippon Sumitomo Pharmaceutical Co., Ltd., Osaka, Japan). HR was calculated from the number of peaks in the pressure waveform.

Data for SBP and HR were analyzed by power spectral analysis, by which they were automatically separated into two frequency bands: a low frequency band (LF: 0.25 - 0.75 Hz) and a high frequency band (HF: 0.75 - 3.0 Hz) [12,20].

### Experimental protocol

After the baseline recording, the experimental protocol was started. STZ and control rats were sequentially assigned to either a hypovolemic group or normovolemic group.

In the hypovolemic group, a 1 ml syringe was connected to the femoral arterial catheter. Blood was manually withdrawn via a syringe from femoral arterial line for a total volume of 6% of ETBV (about 1.2 ml). ETBV was calculated using a previously reported equation for estimating rat blood volume: 0.06 ml/g ×body weight (g) + 0.77 [21]. Each rat was bled in six cycles, with each cycle followed by a 20-minute observation period. Hemodynamic and autonomic recordings were obtained 5 min (acute phase) and 10 min (plateau phase) after each controlled hemorrhage.

In the normovolemic group, each rat was given the same volume of dextran as the blood withdrawal for 15 minutes after each blood withdrawal. This procedure was repeated 6 times to produce normovolemic hemodilution. Hemodynamic and autonomic recordings were obtained 5 min (acute phase) and 10 min (plateau phase) after each dextran infusion.

Arterial blood was analyzed to determine pH, PaCO_2_, PaO_2_, base excess, bicarbonate level and hemoglobin concentration using Rapid Lob 860 (Siemense) at each stage of blood removal. The respiratory rate was also measured by observation.

### Baroreflex Sensitivity (BRS)

In an additional group of STZ and control rats, BRS was explored using the conventional Oxford method [22,23]. After 30 minutes of baseline recording, each rat received a single intravenous bolus injection (0.1 ml) of phenylephrine (0.2 - 2.7 μg/kg) or sodium nitroprusside (0.8 - 27.0 μg/kg) to increase and reduce SBP by 15 - 30 mmHg, respectively. The pressor test was always performed first. Typically, a 30 minute stabilization period between the pressure and depressor test allowed SBP and HR to return to the pretest values ± 5%. The maximal response of SBP to phenylephrine or sodium nitroprusside and the corresponding maximal reflex change in pulse interval were plotted for each drug. BRS was expressed by the slope of the linear regression calculated based on the reflex change in the pulse interval to a given change in SBP.

### Statistical analysis

Results are expressed as the mean ± SD throughout the article. Data analyses were carried out using SPSS for Windows. Statistical analyses were performed using the unpaired *t*-test for comparisons of baseline values between two groups. ANOVA for repeated measures followed by Dunnet's test were used for comparisons of group differences. A p value of less than 0.05 was considered statistically significant.

## Results

### Baseline values in STZ and control rats (Table 1, Figure 1, 2, 3, 4 and 5)

**Table 1:** Changes in Blood Gas, Hemoglobin, Respiratory Rate in STZ and Control Rats during Acute Hemorrhage

		baseline	6% ETBV	12% ETBV	18% ETBV	24% ETBV	30% ETBV	36% ETBV
			loss	loss	loss	loss	loss	loss
Hb (g/dL)	STZ	12.9 ± 0.6 #	12.8 ± 0.8	11.4 ± 0.3*	10.1 ± 0.5*	9.7 ± 0.6*	9.3 ± 0.9*	8.8 ± 0.8*
	Control	14.1 ± 1.2	13.8 ± 0.7	11.6 ± 1.1*	11.6 ± 1.0*	10.2 ± 0.9*	9.5 ± 0.6*	8.9 ± 0.7*

pH	STZ	7.36 ± 0.03 #	7.36 ± 0.01	7.37 ± 0.03	7.36 ± 0.03	7.37 ± 0.02	7.36 ± 0.01	7.35 ± 0.02*
	Control	7.41 ± 0.04	7.42 ± 0.01	7.41 ± 0.02	7.42 ± 0.02	7.41 ± 0.02	7.41 ± 0.02	7.38 ± 0.01*

P_a_CO_2_ (mmHg)	STZ	35.7 ± 0.9 #.	37.2 ± 2.4	34.2 ± 4.2	29.8 ± 1.5*	28.4 ± 1.5*	29.4 ± 1.7*	26.7 ± 2.8*
	Control	42.5 ± 4.6	39.0 ± 1.6	34.6 ± 2.6*	33.6 ± 4.4*	30.1 ± 4.4*	26.8 ± 5.4*	21.2 ± 3.9*

HCO_3_^-^ (mmol/L)	STZ	21.6 ± 3.1#	21.4 ± 2.2	19.7 ± 1.6	18.5 ± 0.9*	17.5 ± 2.1*	15.8 ± 2.8*	13.6 ± 3.0*
	Control	24.9 ± 2.1	25.1 ± 0.7	22.1 ± 1.3	22.0 ± 1.7	19.4 ± 1.6*	16.5 ± 2.7*	14.9 ± 1.3*

BE (mEq/L)	STZ	-3.4 ± 1.5 #	-3.8 ± 2.2	-3.7 ± 1.2	-3.9 ± 2.3	-2.8 ± 1.4	-5.0 ± 1.9*	-7.2 ± 1.3*
	Control	1.6 ± 1.5	1.1 ± 0.7	-0.4 ± 1.3	-1.2 ± 1.0*	-2.9 ± 1.3*	-4.3 ± 1.4*	-5.3 ± 1.3*

RR (times/nin)	STZ	84 ± 5	90 ± 5	100 ± 8	122 ± 7*	137 ± 16*	139 ± 20*	144 ± 12*
	Control	84 ± 4	86 ± 7	100 ± 6	119 ± 9*	133 ± 18*	141 ± 19*	142 ± 15*

Data are the mean ± SD. STZ rats n = 8, Control rats n = 8

# P < 0.05 STZ rats versus Control rats for baseline values;

* P < 0.05 versus the baseline value

**Figure 1:**
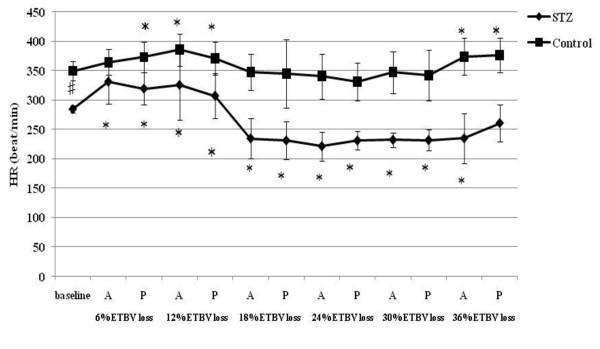
**HR responses to acute hemorrhage**. Data are the mean ± SD. STZ rats n = 8, Control rats n = 8. # P < 0.05 STZ rats versus Control rats for baseline values; * P < 0.05 versus the baseline value A: acute phase, P: plateau phase.

**Figure 2:**
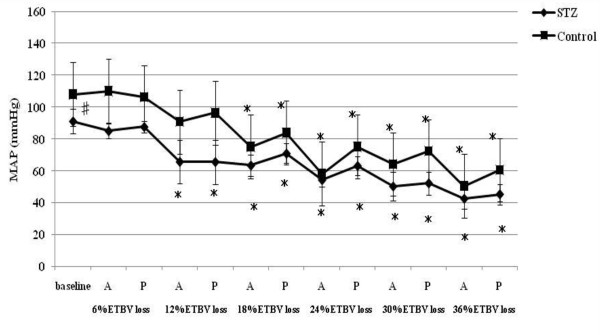
**MAP responses to acute hemorrhage**. Data are the mean ± SD. STZ rats n = 8, Control rats n = 8. # P < 0.05 STZ rats versus Control rats for baseline values; * P < 0.05 versus the baseline value A: acute phase, P: plateau phase.

**Figure 3:**
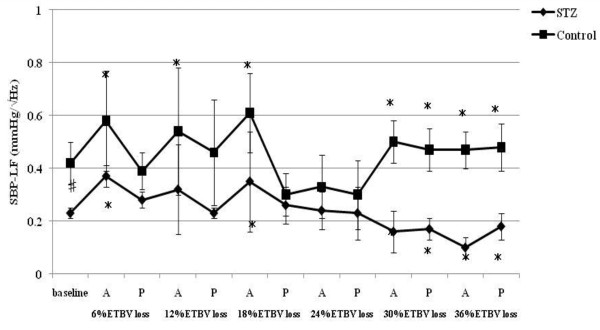
**SBP-LF responses to acute hemorrhage**. Data are the mean ± SD. STZ rats n = 8, Control rats n = 8. # P < 0.05 STZ rats versus Control rats for baseline values; * P < 0.05 versus the baseline value A: acute phase, P: plateau phase.

**Figure 4:**
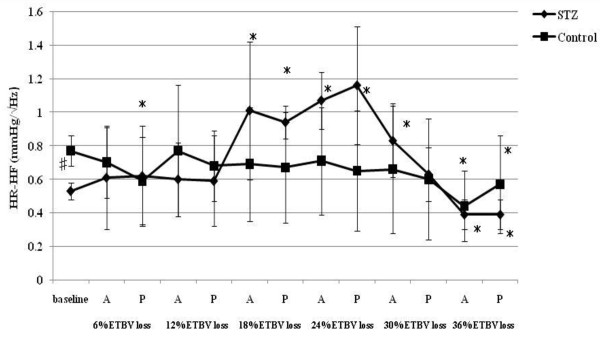
**HR-HF responses to acute hemorrhage**. Data are the mean ± SD. STZ rats n = 8, Control rats n = 8. # P < 0.05 STZ rats versus Control rats for baseline values; * P < 0.05 versus the baseline value A: acute phase, P: plateau phase.

**Figure 5:**
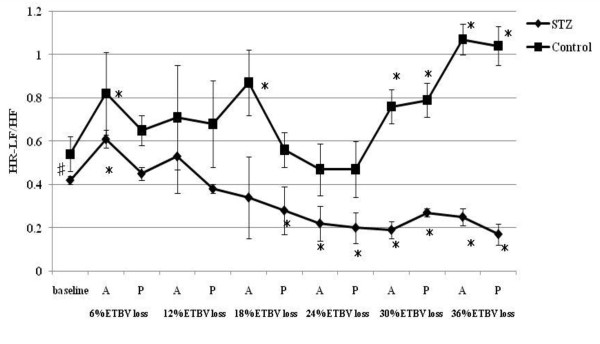
**HR-LF/HF responses to acute hemorrhage**. Data are the mean ± SD. STZ rats n = 8, Control rats n = 8. # P < 0.05 STZ rats versus Control rats for baseline values; * P < 0.05 versus the baseline value A: acute phase, P: plateau phase.

Resting HR, MAP, SBP-LF, HR-HF, HR-LF/HF ratios in STZ rats were lower than those in control rats. The blood glucose level increased to 356.5 ± 12.7 mg/dl in STZ rats. The corresponding value in control rats was 111.1 ± 9.3 mg/dl. The beta hydroxybutyric acid level was also higher in STZ rats (1.1 ± 0.5 mmol/l) than that in control rats (0.0 ± 0.0 mmol/l). The hemoglobin concentration in STZ rats was slightly lower than that in control rats. In arterial blood gas analysis, pH, PaCO_2_, HCO_3_^- ^and base excess in STZ rats were significantly lower than those in control rats. There was no difference in the respiratory rate between STZ and control rats.

BRS was expressed by the slope of the regression line relating to changes between the pulse interval and SBP. The gain in the pressor response due to the administration of phenylephrine was 0.78 ± 0.01 ms/mmHg in STZ rats and 0.98 ± 0.12 ms/mmHg in control rats. There was a significant difference between STZ and control rats. The gain in the depressor response due to sodium nitroprusside was 0.75 ± 0.01 ms/mmHg in STZ rats and 0.96 ± 0.10 ms/mmHg in control rats, respectively. This index in STZ rats was also significantly reduced when compared with control rats.

### Hemodynamic and autonomic responses to acute hemorrhage

A significant decrease in HR was observed in STZ rats between 18% and 30% TEBV loss following a transient increase at 6 and 12% ETBV loss. Although control rats also showed increased HR at 6 and 12% ETBV loss, this response disappeared between 18% and 30% EEBV loss. In STZ rats, a fall in MAP was observed at 12% ETBV loss and this persisted throughout the subsequent blood withdrawal. In control rats, MAP did not decrease until 18% of ETBV was withdrawn. Decompensated hypotension in STZ rats occurred earlier than in control rats.

SBP-LF increased in the acute phase in both rats up to 18% ETBV loss. Although SBP-LF did not show any changes in either group at 24% ETBV loss, when the blood loss volume reached 30% or more ETBV loss, SBP-LF increased again in control rats, while it decreased in STZ rats. HR-HF increased significantly between 18 and 30% ETBV loss in STZ rats. On the other hand, in control rats, HR-HF remained unchanged between 12 and 30% ETBV loss after a transient reduction in the plateau phase at 6% ETBV loss. Finally, both rats showed reduced HR-HF at 36% ETBV loss. In STZ rats, HR-LF/HF increased transiently at 6% ETBV loss and fell after 18% or more ETBV loss. In control rats, an elevation in HR-LF/HF was observed in the acute phase at 6 and 18% ETBV loss and persisted after 30% or more of ETBV was withdrawn.

### Effects on respiratory rate, arterial blood gas analysis and hemoglobin concentration

The respiratory rate significantly increased in both STZ and control rats at 18% ETBV loss when compared to the baseline value. The increase in respiratory rate speeded up as the blood loss volume increased. Therefore, PaCO_2 _significantly decreased in both groups of rats. Hemoglobin concentration was significantly reduced when blood loss volume reached 12% or more of ETBV in both groups of rats; however, there were no significant differences.

### The effects of dextran treatment after hemorrhage (Tables 2, Figure 6, 7, 8, 9 and 10)

**Table 2:** Changes in Blood Gas, Hemoglobin, Respiratory Rate in STZ and Control rats during Normovolemic Hemodilution

		baseline	1st	2nd	3nd	4th	5th	6th
			exchange	exchange	exchange	exchange	exchange	exchange
Hb (g/dL)	STZ	13.4 ± 1.2#	12.1 ± 0.2	11.6 ± 0.4	10.2 ± 1.2*	9.2 ± 0.1*	9.2 ± 0.5*	8.4 ± 0.1
	Control	13.6 ± 1.2	12.8 ± 0.5	11.8 ± 1.2	10.2 ± 0.3*	9.5 ± 1.2*	9.0 ± 0.8*	8.2 ± 0.4*

pH	STZ	7.36 ± 0.03#	7.35 ± 0.03	7.37 ± 0.03	7.37 ± 0.01	7.37 ± 0.03	7.35 ± 0.02	7.35 ± 0.01
	Control	7.44 ± 0.02	7.44 ± 0.03	7.43 ± 0.03	7.42 ± 0.01	7.43 ± 0.01	7.42 ± 0.01	7.39 ± 0.02*

P_a_CO_2_ (mmHg)	STZ	37.4 ± 5.4#	37.0 ± 4.1	33.7 ± 5.0	30.5 ± 3.3*	28.4 ± 2.1*	30.0 ± 2.1*	26.6 ± 2.5*
	Control	41.4 ± 3.4	35.9 ± 2.1	30.8 ± 2.4*	31.5 ± 2.7*	29.1 ± 0.9*	27.0 ± 1.8*	25.9 ± 2.8*

HCO_3_^-^ (mmol/L)	STZ	22.5 ± 1.8#	21.7 ± 0.5	19.8 ± 1.3	18.3 ± 1.2*	18.0 ± 0.8*	16.6 ± 0.8*	13.6 ± 3.0*
	Control	21.1 ± 1.6	19.81 ± 1.8	19.3 ± 1.0	19.4 ± 1.5	20.0 ± 0.7	19.6 ± 1.7	19.9 ± +1.3

BE' (mEq/L)	STZ	-4.1 ± 1.0#	-4.3 ± 0.8	-4.5 ± 1.5	-4.6 ± 1.2	-4.8 ± 1.8	-6.0 ± 1.5*	-6.9 ± 1.2*
	Control	0.8 ± 0.4	-0.8 ± 2.5	-2.3 ± 2.0*	-2.2 ± 2.0*	-2.4. ± 1.7*	-2.3 ± 2.6*	-1.3 ± 2.4*

RR (times/nin)	STZ	85 ± 2	107 ± 15*	118 ± 16*	129 ± 10*	143 ± 14*	140 ± 6*	142 ± 11*
	Control	83 ± 5	88 ± 6	111 ± 8*	118 ± 7*	110 ± 11*	108 ± 11*	116 ± 19*

Data are the mean ± SD. STZ rats n = 8, Control rats n = 8

# P < 0.05 STZ rats versus Control rats for baseline values;

* P < 0.05 versus the baseline value

**Figure 6:**
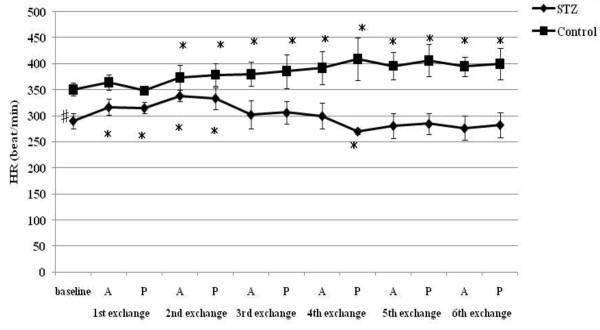
**The effects of dextran treatment to HR after hemorrhage**. Data are the mean ± SD. STZ rats n = 8, Control rats n = 8. # P < 0.05 STZ rats versus Control rats for baseline values; * P < 0.05 versus the baseline Value A: acute phase, P: plateau phase.

**Figure 7:**
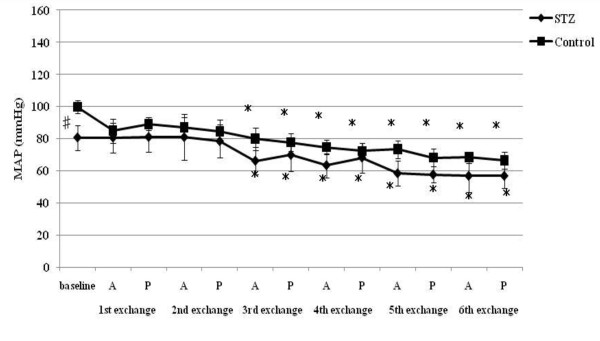
**The effects of dextran treatment to MAP after hemorrhage**. Data are the mean ± SD. STZ rats n = 8, Control rats n = 8. # P < 0.05 STZ rats versus Control rats for baseline values; * P < 0.05 versus the baseline Value A: acute phase, P: plateau phase.

**Figure 8:**
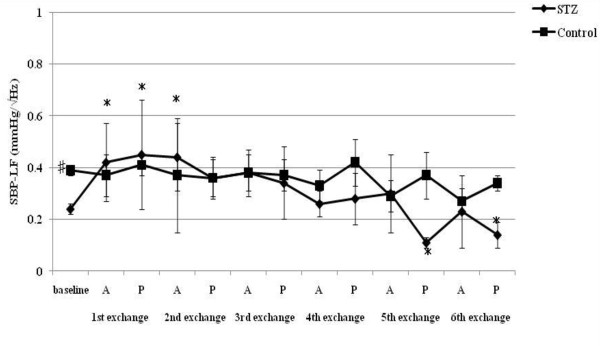
**The effects of dextran treatment to SBP-LF after hemorrhage**. Data are the mean ± SD. STZ rats n = 8, Control rats n = 8. # P < 0.05 STZ rats versus Control rats for baseline values; * P < 0.05 versus the baseline Value A: acute phase, P: plateau phase

**Figure 9:**
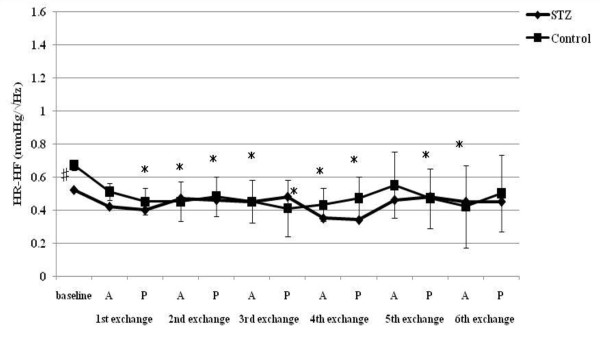
**The effects of dextran treatment to HR-HF after hemorrhage**. Data are the mean ± SD. STZ rats n = 8, Control rats n = 8. # P < 0.05 STZ rats versus Control rats for baseline values; * P < 0.05 versus the baseline Value A: acute phase, P: plateau phase.

**Figure 10:**
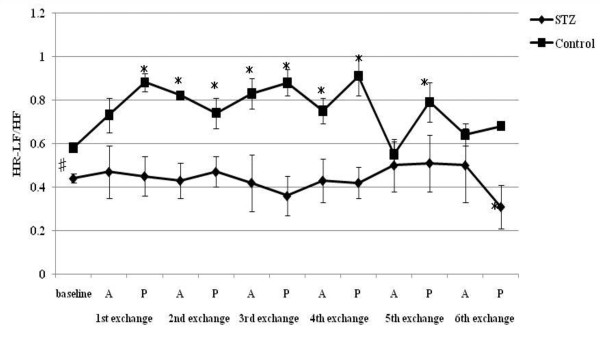
**The effects of dextran treatment to HR-LF/HF after hemorrhage**. Data are the mean ± SD. STZ rats n = 8, Control rats n = 8. # P < 0.05 STZ rats versus Control rats for baseline values; * P < 0.05 versus the baseline Value A: acute phase, P: plateau phase.

In both groups of rats, hemoglobin concentrations were significantly reduced after the third blood withdrawal and dextran treatment as compared with the baseline values. Therefore, hemodilution occurred after the third exchange in both rats.

HR increased significantly after the first and second exchange in STZ rats. In control rats, a significant increase in HR was sustained after the second exchange. A reduction in MAP was observed after the third exchange in both rats. SBP-LF increased transiently after the first and second exchange and finally decreased in STZ rats. However, there were no significant changes in SBP-LF in control rats. STZ rats showed no significant changes in HR-HF during normovolemic hemodilution, while control rats showed a significant reduction in HR-HF. HR-LF/HF increased in control rats between the first and fifth exchange, while remaining unchanged in STZ rats.

An increased respiratory rate was observed after the first exchange in STZ rats and after the second in control rats.

## Discussion

To our knowledge, the present study is the first to evaluate the effects of STZ-induced diabetic cardiovascular autonomic neuropathy during acute hemorrhage using BP-HR variability analysis. There were two major findings in this study. First, STZ rats showed different autonomic and hemodynamic responses to acute hemorrhage from control rats. During moderate hemorrhage (18 -30% ETBV loss), the decrease in HR was prominent in STZ rats due to the activation of parasympathetic drive, as indicated by the increase in HR-HF, while in control rats this response was not observed. The second major finding was that the administration of dextran prevented the activation of parasympathetic drive in STZ rats during moderate hemorrhage. Furthermore, in control rats, dextran treatment sustained the initial increase in HR with reduced HR-HF throughout the course of the hemorrhage.

### Hemodynamic and autonomic alterations in STZ rats

In experimental studies, administration of STZ is a well-established method for inducting diabetes in rats. This model has been commonly used to study the pathogenic mechanisms of various complications associated with diabetes, including autonomic dysfunction. In the present study, the resting levels of BP and HR were reduced in STZ rats compared with control rats. These results were consistent with previous studies [24,25]. The mechanisms underlying the hemodynamic changes observed in STZ rats are unknown. However, because impaired BRS and a reduced power spectrum of BP-HR variability have been shown in the same experimental model [24-28], autonomic alteration is responsible at least in part for hemodynamic changes in STZ rats. In addition, the reduced HR may reflect changes in the electrophysiological properties of the sino-atrial node [29,30].

There is controversy regarding the alterations in baroreflex control of HR in diabetes-induced models. Maeda et al. reported that the tachycardiac reflex response elicited by a reduction in arterial pressure was attenuated, while the bradycardiac reflex response to increased arterial pressure was normal, 5 days after STZ administration [31]. In the present study, we observed attenuation of both bradycardiac and tachycardiac reflex responses to blood pressure changes 14 days after STZ administration. Our results were similar to the results of Carmo et al. [32], indicating that diabetic rats were associated with impaired BRS as early as 7 days after STZ administration. On the other hand, Chang et al. demonstrated that baroreflex sensitivity increased 12 weeks after STZ treatment and decreased 48 weeks later [33]. According to these results, baroreflex-mediated HR changes appear to be dependent on the time course of the disease.

### Responses to blood withdrawal in STZ rats

A variety of methods have been used to induce a hypovolemic state in previous experiments. They include methods such as withdrawing blood at a fixed rate [34,35], bleeding to a fixed arterial blood pressure [36], a fixed volume of withdrawal [37], and a lower body negative pressure [38]. The method used to induce hypovolemia can have a huge impact on hemodynamic and autonomic responses. Even if the total volume of blood loss is the same, different results may be obtained depending on the method of blood withdrawal. In the present study, a controlled graded bleeding was performed with 20-minute intervals between two withdrawals [39]. This method makes it possible to measure the autonomic and hemodynamic response to each volume of blood loss. BP-HR variability measured in the plateau phase (10 minutes after withdrawal) reflects the steady state condition after each blood loss, leaving ample time for circulatory compensation.

During mild blood loss (6-12% ETBV loss), an increase in HR was observed in control rats. This response was associated with a transient reduction in parasympathetic drive and activation of sympathetic drive, as indicated by changes in HR-HF power and in HR-LF/HF and SBP-LF power, respectively. As blood loss approached a moderate level (18-30% ETBV of loss), the initial increase in HR disappeared and a significant reduction in MAP was noted. However, BP-HR variability did not show any marked changes in this phase. As the hemorrhage progressed further (more than 30% ETBV loss), a marked reduction in MAP developed. In the present study, control rats did not show the sympatho-inhibitory phase of acute hemorrhage characterized by decreased HR associated with elevated parasympathetic drive to the heart. This inhibitory response is mediated by the B-J reflex. Baujard et al. showed similar results to ours when using a controlled graded hemorrhage [39]. Porter et al. demonstrated that hemorrhage at a slow rate (20 ml/kg/40 min) did not cause elevation in HR-HF [40]. Conversely, hemorrhage at an intermediate or fast rate induced a decrease in HR associated with a significant increase in HF power [41]. These results suggest that the rate of hemorrhage has an important impact on autonomic regulation. Because the method of blood loss used in the present study may have been equivalent to a slow rate hemorrhage, the sympatho-inhibitory phase of acute hemorrhage was not manifested in control rats. Kawase et al. reported that massive hemorrhage of MAP of 50 mmHg caused two types of response in anesthetized dogs: bradycardia followed by tachycardia and continuous tachycardia [42]. The HR response to hemorrhage may also be dependent on the anesthetic condition or species, as well as the rate of hemorrhage.

On the other hand, STZ rats showed significant bradycardia associated with the activation of parasympathetic drive and reduction in sympathetic drive indicated by HR variability during moderate hemorrhage, despite the same rate of blood loss as control rats. This different response to hemorrhage between STZ and control rats suggests autonomic alteration induced by diabetic neuropathy. The enhancement of the B-J reflex in STZ rats may reflect the autonomic disterbance. The B-J reflex, as well as the arterial baroreflex and cardiopulmonary reflex, constitute a short-term regulatory system for maintaining cardiovascular stability. The B-J reflex is elicited by the stimulation of left ventricular mechanoreceptors and its signal reaches the central autonomic network via unmyelinated vagal afferent fibers. Although the B-J reflex has a common afferent pathway with the aortic baroreflex, the signals from left ventricular mechanoreceptors connect to a different site of the brainstem from that of the arterial baroreceptor [43]. The activation of the brainstem by the B-J reflex induces an increase in parasympathetic tone to the heart and the withdrawal of sympathetic nervous activity. Previous studies indicated that the B-J reflex blunted the arterial baroreflex due to interference with the baroreflex signal transduction in the brainstem [44].

Impairment of baroreflex function due to diabetes has been reported [31-33]. Although the exact site of the defect in the reflex arch has not been determined, it could involve sensory baroreceptor endings, the afferent pathway, connections within the central autonomic network, the efferent component of the reflex pathway, or altered muscarinic receptors to acetylcholine in the heart. The present study showed a reduction in the HR response to blood pressure changes due to the administration of phenylephrine or nitroprusside in STZ rats. However, Dall'Ago et al. demonstrated that the bradycardia elicited by electrical stimulation of vagal efferents induced a greater HR response in diabetic rats compared to controls [45]. They also observed greater bradycardia in response to methacholine in diabetic rats. Homma et al. studied HR and BP responses to the stimulation of peripheral cut ends of cervical sympathetic and vagus nerves in diabetic rats and showed that the HR response was exaggerated and that the BP response was disturbed in diabetic rats [46]. Furthermore, the effects of diabetes on the central nervous system are minimal [47] and diabetic autonomic neuropathy primarily involves the peripheral autonomic nerve. These findings suggest that the efferent pathway of the baroreflex is not responsible for the reduced BRS in diabetic rats.

A reduction in arterial BP and stretching of the cardiac wall due to massive blood loss reduces the discharge from the arterial baroreceptor and increases the discharge from the left ventricular mechanoreceptor, respectively. Although deactivation of the arterial receptor and activation of the mechanoreceptor elicit the opposite effects on sympathetic nerves and the parasympathetic nervous system, the signals from these receptors are modulated through the central autonomic network and then the final reflex response develops in the cardiovascular system. Diabetes impairs the arterial baroreflex, but not the B-J reflex, according to the results of the present study. Not all the circulatory reflexes are impaired equally by diabetes. This finding is supported by the fact that the impairments in vascular reactivity to vasoconstrictors due to diabetes vary greatly depending on the site of artery or gender [48-50]. Because the afferent pathway of the arterial baroreflex seems to be easily influenced by diabetic neuropathy, the hemodynamic response to the B-J reflex may be enhanced and consequently, hypotension with bradycardia is manifested in STZ rats.

The B-J reflex has a beneficial effect on the circulatory system under normal circumstances. Kawase et al. reported that massive hemorrhage caused two types of HR response: bradycardia and tachycardia, in anesthetized dogs, and that tachycardiac dogs may have a worse outcome than bradycardiac dogs [42]. Batchinsky et al. also showed that a characteristic change in HR variability was sympathetic withdrawal due to the B-J reflex in all surviving subjects after severe hemorrhage [51]. On the other hand, Barnas et al. demonstrated that activation of the B-J reflex was involved in severe symptomatic hypotension accompanied by bradycardia during dialysis and that half of the patients who were prone to dialysis hypotension had a history of diabetes [52]. This finding suggests that diabetic neuropathy is involved in enhancement of the B-J reflex. The cardiodepressor response due to the enhanced B-J reflex may cause autonomic disturbance in patients with diabetic autonomic neuropathy. Excess activation of the B-J reflex may lead to severe bradycardiac hypotension, placing the patient's life at risk.

### The effects of dextan treatment

In STZ rats, the administration of dextran after blood withdrawal prevented the activation of parasympathetic drive, as indicated by an increased HR-HF. As a result, bradycardiac hypotension did not develop in STZ rats. Maintaining cardiac filling with the administration of dextran inhibited the B-J reflex. The autonomic response to dextran treatment in control rats was characterized by reduced parasympathetic activity and sympathetic predominance. Therefore, the initial increase in HR was sustained and a reduction in MAP did not occur until the third hemodilution. Tanaka et al. reported that arterial baroreflex control of HR was not altered during profound acute normovolemic hemodilution to a hemoglobin concentration of 4-5 g/dl using a phenylephrine pressor and nitroprusside depressor test in pentobarbital-anesthetized dogs [53].

### Limitations

There are several limitations to this study. First, there is a discrepancy in the autonomic condition between STZ-induced diabetic rats and human diabetic patients. The results of the present study demonstrated that STZ-induced diabetes was associated with bradycardia. Our results are in agreement with the data from other laboratories [24-28]. On the other hand, the majority of human clinical studies reported tachycardia. Many investigators believe that the decline in parasympathetic function precedes the impairment in sympathetic nervous control in diabetic patients [54,55].

Observed differences between STZ-induced diabetic rats and patients in clinical situations may result from species variation or from differences inherent in pathogenic mechanisms. The induction of diabetes in the rat results from a single insult to pancreatic beta-cells and immediate and sustained hyperglycemia develops. On the other hand, in humans the cause and time of onset of diabetes are usually unknown and the time course of the rise in blood glucose less well defined.

Second, the change in respiration may have a profound impact on HR variability parameters. In fact, the HR-HF power increases as tidal volume increases, independent of changes in parasympathetic drive [56,57]. Also, an increase in the respiratory rate could reduce the HR-HF power [58,59]. In the present study, we measured only the respiratory rate, but not tidal volume. Therefore, it is impossible to precisely evaluate the effects of respiratory control on HR variability. However, the reduction in HR-HF was not observed in control rats until 36% of ETBV was withdrawn, despite a consistent increase in respiratory rate after hemorrhage. In addition, STZ rats showed a decrease in HR-HF during severe hemorrhage despite an increase in the respiratory rate. These findings suggest that the difference in HF power observed during the hemorrhage reflected increases in parasympathetic drive, rather than changes in respiratory control induced by hemorrhage. It is impossible to explain the changes in HR-HF observed in the present study only on the basis of the change in respiratory control.

## Conclusions

In summary, our study demonstrated that rats with diabetic neuropathy were sensitive to developing bradycardiac hypotension during acute hemorrhage. The autonomic responses were characterized by marked parasympathetic activation. These findings suggest enhancement of the B-J reflex in diabetic rats. On the other hand, dextran treatment inhibited the B-J reflex. The mechanisms for an enhanced B-J reflex in diabetic rats are unknown and future studies are needed to better identify them.

## Abbreviations

BP: Blood pressure; B-J reflex: Bezold-Jarisch reflex; BRS: Baroreflex Sensitivity; CAN: Cardiovasucular Autonomic Neuropathy; ETBV: Estimated Total Blood Volume; HF: High Frequency; HR: Heart Rate; HR-HF: The HF component of HR; HR-LF/HF: The ratio of LF to HF in HR; LF: Low Frequency; MAP: Mean Arterial Pressure; SBP: Systolic Blood Pressure; SBP-LF: The LF component of SBP; STZ-rats: Streptozotocin-induced diabetic rats

## Competing interests

The authors declare that they have no competing interests.

## Authors' contributions

All authors contributed equally to this work. All authors contributed to the design of the study, interpretation of the results, and critical revision of the manuscript. All authors have read and approved the final manuscript.

## Authors' information

AB: Dental Anesthesiologist, D.D.S, Ph.D., Postdoctoral Fellow

MS: Dental Anesthesiologist, D.D.S, Ph.D., Associate Professor

YM: Dental Anesthesiologist, D.D.S, Ph.D., Associate Professor

HH: Dental Anesthesiologist, D.D.S, Ph.D., Assistant Professor

HN: Dental Anesthesiologist, D.D.S, Ph.D., Professor
